# Stroke alters behavior of human skin-derived neural progenitors after transplantation adjacent to neurogenic area in rat brain

**DOI:** 10.1186/s13287-017-0513-6

**Published:** 2017-03-09

**Authors:** Carlos de la Rosa-Prieto, Cecilia Laterza, Ana Gonzalez-Ramos, Somsak Wattananit, Ruimin Ge, Olle Lindvall, Daniel Tornero, Zaal Kokaia

**Affiliations:** 1 Laboratory of Stem Cells and Restorative Neurology, Lund Stem Cell Center, University Hospital, 221 84 Lund, Sweden; 2 Present address: Laboratory of Human Neuroanatomy, Department of Health Sciences, Faculty of Medicine, CRIB, University of Castilla-La Mancha, 02008 Albacete, Spain

**Keywords:** Adult neurogenesis, Human skin-derived cells, Pluripotent, Rostral migratory stream, Stroke, Subventricular zone, Transplantation

## Abstract

**Background:**

Intracerebral transplantation of human induced pluripotent stem cells (iPSCs) can ameliorate behavioral deficits in animal models of stroke. How the ischemic lesion affects the survival of the transplanted cells, their proliferation, migration, differentiation, and function is only partly understood.

**Methods:**

Here we have assessed the influence of the stroke-induced injury on grafts of human skin iPSCs-derived long-term neuroepithelial-like stem cells using transplantation into the rostral migratory stream (RMS), adjacent to the neurogenic subventricular zone, in adult rats as a model system.

**Results:**

We show that the occurrence of an ischemic lesion, induced by middle cerebral artery occlusion, in the striatum close to the transplant does not alter the survival, proliferation, or generation of neuroblasts or mature neurons or astrocytes from the grafted progenitors. In contrast, the migration and axonal projection patterns of the transplanted cells are markedly influenced. In the intact brain, the grafted cells send many fibers to the main olfactory bulb through the RMS and a few of them migrate in the same direction, reaching the first one third of this pathway. In the stroke-injured brain, on the other hand, the grafted cells only migrate toward the ischemic lesion and virtually no axonal outgrowth is observed in the RMS.

**Conclusions:**

Our findings indicate that signals released from the stroke-injured area regulate the migration of and fiber outgrowth from grafted human skin-derived neural progenitors and overcome the influence on these cellular properties exerted by the neurogenic area/RMS in the intact brain.

## Background

Intracerebral transplantation of induced pluripotent stem cells (iPSCs) or their derivatives, generated by reprogramming human somatic cells, can reverse behavioral deficits in experimental stroke models (for references, see [1]). Improvements were detected early after transplantation, indicating that they were not due to neuronal replacement but to other mechanisms such as modulation of inflammation, and promotion of plastic responses and neovascularization. However, we recently showed that human iPSC-derived long-term neuroepithelial-like stem cells (lt-NESCs) transplanted into stroke-injured cortex can differentiate to form functional cortical neurons, which receive afferent inputs from appropriate brain areas and respond to mechanical stimulation of nose and paw [2]. It remains to be demonstrated, though, that this incorporation into host neural circuitry contributes to the long-term functional recovery after stroke.

A fundamental question, also in a clinical-therapeutical perspective, is how the stroke-induced injury affects the survival, proliferation, migration, neuronal differentiation, integration and function of the grafted human iPSC-derived cells. Implantation of the cells in the rostral migratory stream (RMS), close to one of the main neurogenic areas in the brain, the subventricular zone (SVZ), seems to be a useful model system to address these issues. In the SVZ, neural stem/progenitor cells form new neurons that travel, via the RMS, toward the main olfactory bulb (MOB) [3], where they integrate as interneurons in the granule and periglomerular cell layers. Several cues have been identified which take part in this process [4–6] and may regulate the behavior also of cells transplanted close to the SVZ into the adult rodent brain. For example, differentiated neurons from a human teratocarcinoma send their axons through the RMS and in association with ipsilateral and contralateral white matter pathways [7]. When implanting human skin-derived iPSCs close to the SVZ in adult intact brains, most of the grafted cells migrate via the RMS. In contrast, oligodendrocytes implanted do not migrate along the RMS but follow the white matter pathways along the corpus callosum and internal capsule [8]. Whether the occurrence of an ischemic lesion affects the migration, axonal outgrowth or other properties of human iPSCs implanted close to the SVZ is unknown.

Here we have explored the role of the ischemic injury for the behavior of human iPSC-derived lt-NESCs after transplantation into the RMS, adjacent to the neurogenic SVZ, in intact and stroke-damaged rats. We demonstrate that the stroke-induced lesion markedly alters the patterns of axonal outgrowth and migration of the grafted cells, whereas survival, proliferation, and generation of neuroblasts, mature neurons or astrocytes are unaffected.

## Methods

### Animals

Male (225–250 g) Sprague-Dawley (SD) rats (n = 16; Charles River Laboratories Wilmington, MA, USA) were used. They were housed in standard caging under a 12-hour light/dark cycle with ad libitum access to food and water.

### Cell culture

The human iPSC-derived lt-NESCs were produced from human skin fibroblasts as previously described [9]. Briefly, human fibroblasts were subjected to retroviral transduction with plasmids encoding for the viral glycoprotein VSV-G and the reprogramming factors Oct4, Sox2, KLF4, and c-MYC and split into plates with mouse embryonic fibroblasts. Colonies were then picked and expanded to establish human iPSC lines. Those lines were induced to differentiate to neural phenotype as previously described [10] through an embryoid body-production step. Neural rosettes were generated and carefully picked and grown in the presence of 10 ng/ml fibroblast growth factor (FGF) 2, 10 ng/ml epidermal growth factor (EGF; both from R&D Systems, Inc., Minneapolis, MN, USA), and 1 μl/ml B27 (Invitrogen, Carlsbad, CA, USA). The human iPSC-derived lt-NES cell line was routinely cultured and expanded on 0.1 mg/ml poly-L-ornithine- and 10 μg/ml laminin- (both from Sigma-Aldrich, St. Louis, MO, USA) coated plates in the same media supplemented with FGF, EGF and B27. The lt-NESCs were passaged at a ratio of 1:2 to 1:3 every second to third day using trypsin (Sigma-Aldrich).

### Middle cerebral artery occlusion

Stroke was induced using the intraluminal filament model of middle cerebral artery occlusion (MCAO) as previously described [11, 12]. Briefly, right common carotid artery (CCA) and its proximal branches were isolated. The CCA and external carotid artery (ECA) were ligated, and internal carotid artery (ICA) was temporarily occluded using a metal micro-vessel clip. A nylon monofilament was advanced through the CCA and ICA until resistance was felt (approximately 9 mm distance) past the origin of the middle cerebral artery. The nylon filament was carefully removed after 30 minutes occlusion, the ECA was ligated permanently, and the surgical wound was closed. Special care was taken for a week after surgery. A high-calorie gel diet (DietGel™ Boost, ClearH2O, Westbrook, ME, USA) was supplemented and Ringer’s solution was injected subcutaneously daily in case of dehydration.

### Transplantation

Intracerebral transplantation of human iPSC-derived lt-NESCs, which previously had been transduced with lentivirus carrying green fluorescent protein (GFP), was performed stereotaxically. At the day of surgery, cells were resuspended to a final concentration of 10^5^ cells/μl. A volume of 2 μl was injected at the following coordinates (from bregma and brain surface): anterior/posterior (AP): +1.8 mm; medial/lateral (M/L): +1.7 mm; dorsal/ventral (D/V): -4 mm. Tooth bar was set at -3.3 mm. Rats were given subcutaneous injections of 10 mg/kg Cyclosporine A every day during the first month after transplantation and every other day during the second month.

### Immunohistochemistry

Rats were sacrificed and perfused transcardially with 4% paraformaldehyde. Sagittal sections (30 μm) of fixed brains obtained with a microtome were preincubated in blocking solution (5% normal serum and 0.25% Triton X-100 in 0.1 M potassium phosphate-buffered saline). Sections were incubated at +4 °C overnight with primary antibodies diluted in blocking solution. The following antibodies were used: chicken anti-green fluorescent protein (GFP; 1:5000 EMD Millipore, Billerica, MA, USA), mouse anti-NeuN (1:500 EMD Millipore), mouse anti-human cytoplasm SC121 (1:200 StemCell Technologies, Vancouver, BC, Canada), mouse anti-human nuclei SC101 (1:500 StemCell Technologies), rabbit anti-Ki67 (1:400 Novacastra Laboratories, Newcastle, UK), mouse anti-human glial fibrillary acidic protein (GFAP; 1:500 Stem Cell Technologies), rabbit anti-kidney-type glutaminase (KGA; 1:200 Abcam, Cambridge, MA, USA), goat anti-doublecortin (DCX; 1:400 Santa Cruz Biotechnologies, Dallas, TX, USA), and rabbit anti-GAD65/67 (1:400 Sigma-Aldrich). Primary antibodies were detected with appropriate fluorescent or biotinylated secondary antibodies (1:200 The Jackson Laboratories, Bar Harbor, ME USA). Hoechst 33342 (1:4000 Invitrogen) was used to label cell nuclei.

### Quantifications

Numbers of cells immunoreactive for the different markers were estimated stereologically using C.A.S.T.-Grid software (Visiopharm, Hørsholm, Denmark). Around 500 cells per animal were counted in a predefined fraction of the graft area in an epifluorescence/light microscope. Results for NeuN and Ki67 were expressed as percentage of total number of SC101+ cells. For human-specific GFAP and KGA, the fraction of grafted area (GFP+) immunoreactive for each marker was identified with defined representative ranges of threshold for specific signal using image analysis with CellSens Dimension 2010 software (Olympus, Tokyo, Japan), which calculated the total area covered by pixels/specific immunopositive signal. Colocalization of different markers was in all cases validated in a confocal microscope (Carl Zeiss Microscopy GmbH, Jena, Germany).

To estimate fiber density, GFP+/SC121+ immunostaining was used. All fibers crossing the rostral turn of RMS and fibers arriving to the MOB were counted and compared between groups.

For analysis of migration, all nuclei of grafted cells were located based on SC101 immunostaining. Distance from each grafted cell to the injection site was calculated using ImageJ software. Mean and maximum distances of migration were compared between groups.

### Statistics

Comparisons were performed with Prism 6 software (GraphPad Software, San Diego, CA, USA) using unpaired *t* test. Data are presented as mean ± SEM, and differences considered significant at *P* < 0.05.

## Results

### Stroke does not affect survival, proliferation, or differentiation of human skin-derived neural progenitors transplanted adjacent to subventricular zone

**Fig. 1:**
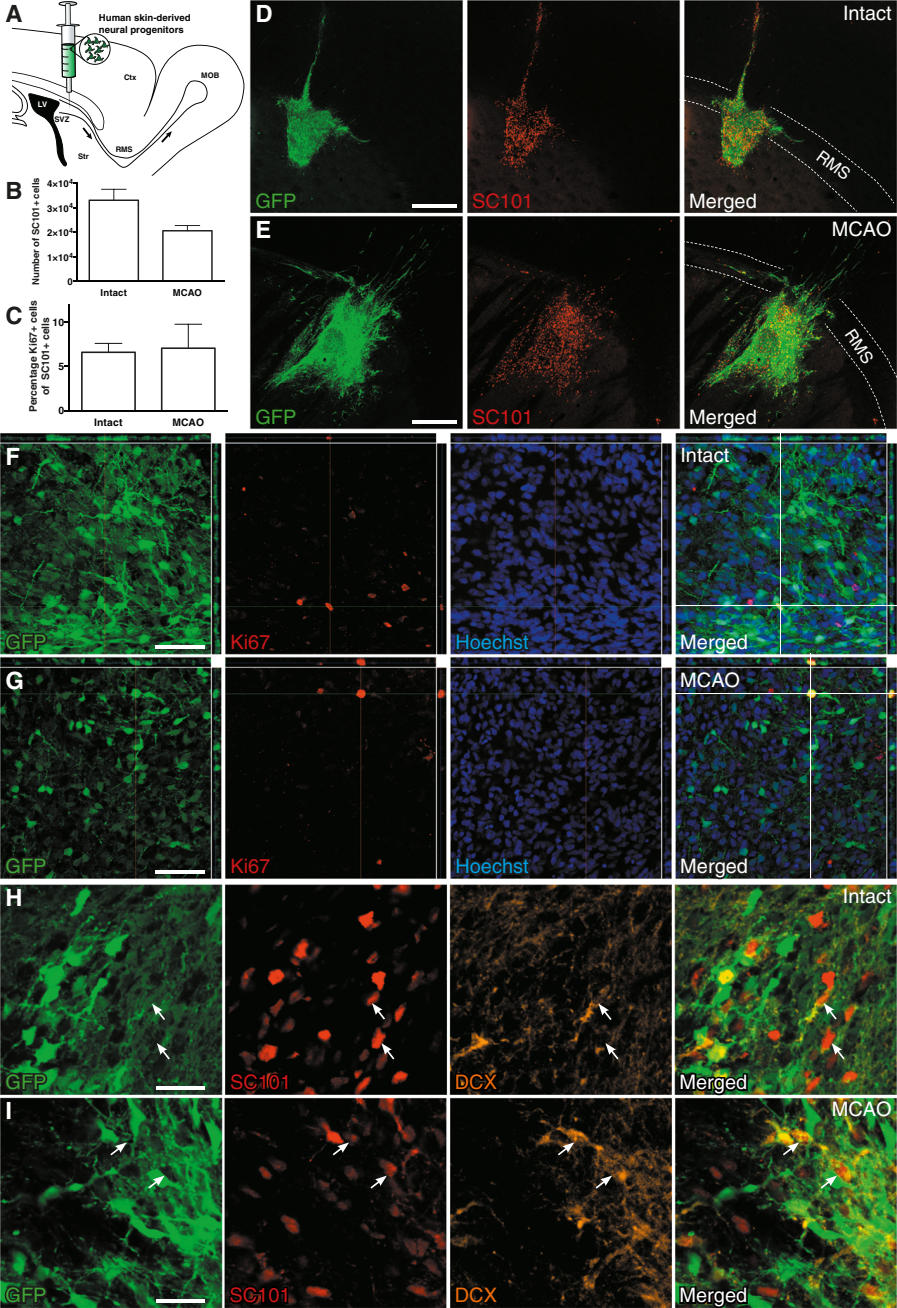
Stroke does not alter survival or proliferation of human skin-derived neural progenitors transplanted adjacent to SVZ. **a** Schematic representation of transplantation approach and injection site in relation to different structures. **b**-**c** Survival (**b**) and proliferation (**c**) of grafted cells in intact (n = 6) and stroke-subjected (MCAO; n = 7) rats at 2 months after transplantation. Data represent means ± SEM. **d-e** Fluorescence photomicrographs showing location of grafted cells (GFP+, *green*) transplanted in the intact (**d**) and stroke-injured (MCAO) rats (**e**) immunostained (in *red*) with human-specific marker SC101, in relation to RMS. **f**-**g** Fluorescence photomicrographs showing examples of grafted cells (GFP+, *green*) transplanted in the intact (**f**) and stroke-injured (MCAO) rats (**g**) co-expressing Ki67 (in *red*). Hoechst nuclear counterstain (in *blue*) is included in (**f-g**). **h**-**i** Fluorescence photomicrographs showing examples of grafted cells (GFP+, *green*) transplanted in the intact (**h**) and stroke-injured (MCAO) rats (**i**) immunostained with SC101 (in *red*) and co-expressing DCX (in *orange*). *Arrows* depict examples of GFP+/SC101+/DCX+ cells. *Ctx* cortex, *LV* lateral ventricle, *SVZ* subventricular zone, *Str* striatum, *RMS* rostral migratory stream, *MOB* main olfactory bulb. Scale bars represent 300 μm in (**d** and **e**), 50 μm in (**f** and **g**) and 25 μm in (**h** and **i**)

In this experiment, eight intact adult rats received transplants of human iPSC-derived lt-NESCs into the RMS, close to the SVZ (Fig. 1a). Another eight rats were subjected to MCAO and after 48 hours were implanted in the same way. Two months later, both groups of animals were sacrificed and perfused, brains were sectioned in the sagittal plane and analyzed by immunohistochemistry.

The transplanted cells were identified using the human-specific nuclear marker SC101. We found that the implantation site, as determined by SC101 staining and localization of the injection track, was situated in the RMS, 0.5 to 1 mm anterior to the lateral ventricle in all animals, without difference between the groups. Using NeuN staining, we then assessed the location of the ischemic damage in the stroke-subjected animals. Neuronal loss was confined to the lateral striatum. The distance from the border of the ischemic injury to the implantation site varied, depending of the extent of the damage, between 1 and 3 mm with an average value of 1.82 mm. There was no significant difference in numbers of grafted cells between stroke-subjected and intact rats at 2 months after transplantation (Fig. 1b and d-e). Similarly, we did not find any difference between the two animal groups in either the numbers of proliferating Ki67+ cells within the grafts (Fig. 1c and f-g) or the percentage of grafted cells immunopositive for the neuroblast marker DCX (59 ± 2.6% and 54.5 ± 4.3% of grafted cells in intact and stroke-injured rats, respectively; Fig. 1h-i).

**Fig. 2:**
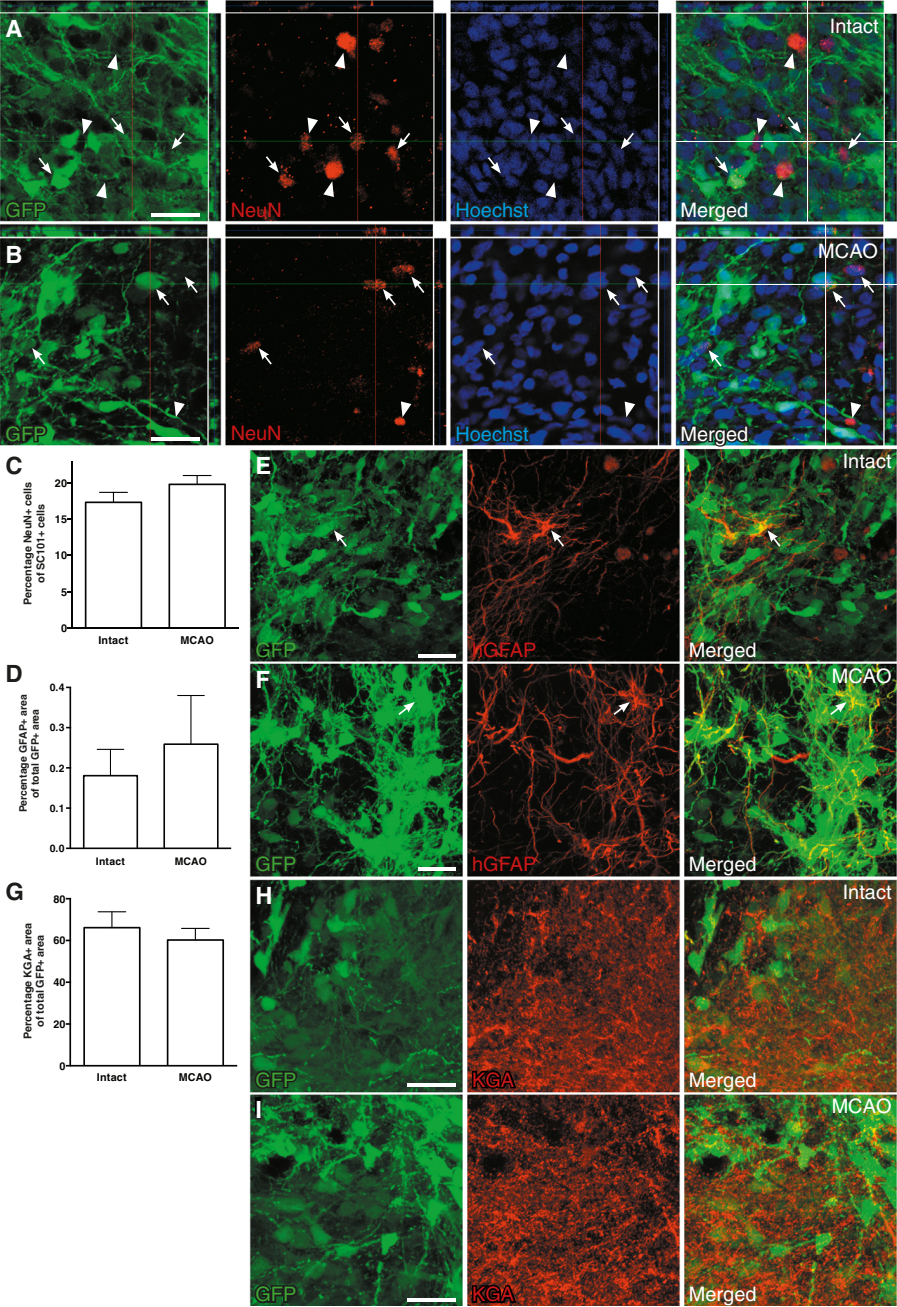
Stroke does not affect differentiation capacity of human skin-derived neural progenitors transplanted adjacent to SVZ. **a**-**b** Fluorescence photomicrographs showing grafted cells (GFP+, *green*) co-expressing the mature neuron-specific marker NeuN (*red*) and Hoechst counterstain (*blue*) 2 months after transplantation in intact (**a**) and stroke-subjected (MCAO, **b**) rats. *Arrows* depict grafted NeuN+ cells while *arrowheads* depict host NeuN+ cells. **c**-**d** Percentage of NeuN+ cells (**c**) and GFAP+ area (**d**) in the grafts from intact (n = 6) and stroke-injured (n = 7) rats. Data represent means ± SEM. Fluorescence photomicrographs showing grafted cells (GFP+, *green*) co-expressing human-specific GFAP (in *red*) 2 months after transplantation in intact (**e**) and stroke-injured (MCAO, **f**) rats. *Arrows* depict grafted GFAP+ cells. **g** Percentage KGA+ area (glutamatergic neuron marker) in grafts from intact (n = 6) and stroke-subjected (n = 7) rats. Data represent means ± SEM. **h** -**i** Fluorescence photomicrographs showing grafted cells (GFP+, *green*) co-expressing KGA (in *red*) 2 months after transplantation in intact (**h**) and stroke-subjected (MCAO, **i**) rats. Scale bars represent 25 μm

We have previously shown that human iPSC-derived lt-NESCs differentiate to mature neurons and, in a small percentage, to mature astrocytes after transplantation into the stroke-injured brain [13, 14]. To determine whether the ischemic lesion affects this differentiation process, we evaluated the capacity of the grafted cells to form mature neurons and astrocytes at 2 months after transplantation into the RMS, close to the SVZ. We found that more than 15% of the grafted cells expressed the mature neuronal marker NeuN when transplanted into the intact brain (16.7 ± 1.6%; Fig. 2a). This percentage did not differ from that found in animals subjected to stroke (19.8 ± 1.2%; Fig. 2b-c). As expected, the proportion of astrocytes immunopositive for human-specific GFAP, generated from the human iPSC-derived lt-NESCs transplanted into the intact brain, was very low at 2 months after transplantation (0.18 ± 0.07% of grafted area covered by GFAP; Fig. 2d and e). The ischemic lesion did not alter this percentage (0.26 ± 0.12%; Fig. 2d and f). Analysis of the phenotype of the neurons generated from the grafted cells showed that the majority of them were positive for the glutamatergic neuron-specific marker KGA with no difference between the groups (66.1 ± 3.8% and 60.2 ± 2.8% of grafted area covered for intact and stroke-subjected animals, respectively; Fig. 2g-i). Accordingly, only few grafted cells were immunopositive for the GABAergic neuron-specific marker GAD65/67 (data not shown).

### Stroke alters migration and axonal projection patterns of human skin-derived neural progenitors transplanted adjacent to subventricular zone

**Fig. 3:**
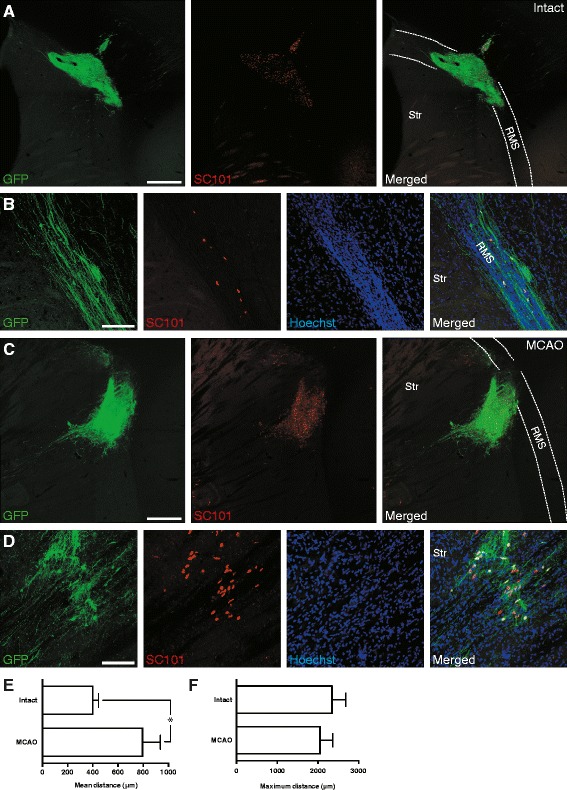
Stroke alters migration pattern of human skin-derived neural progenitors transplanted adjacent to SVZ. **a**-**d** Fluorescence photomicrographs showing migration pattern of grafted cells (GFP+, *green*) 2 months after transplantation in intact (**a**-**b**) and stroke-injured (**c**-**d**) rats. Low magnification overviews (**a**, **c**) and higher magnifications of the migrating grafted cells (**b**, **d**) are presented for each condition. Note the difference in the direction of the migratory pattern of grafted cells between the groups. **e**-**f** Analysis of mean (**e**) and maximum (**f**) distances migrated by the grafted cells from the implantation site at 2 months after transplantation in intact (around 2000 cells analyzed per animal, n = 6) and stroke-injured (around 2000 cells analyzed per animal, n = 7; MCAO) rats. Data represent means ± SEM; *P < 0.05, two-tailed unpaired *t* test. *Str* striatum, *RMS* rostral migratory stream. Scale bars represent 500 μm in (**a** and **c**) and 100 μm in (**b** and **d**)

The human iPSC-derived lt-NESCs implanted near the SVZ in the intact brain showed a selective but limited migration along the descending limb of the RMS (for definition, see e.g., [15]). Thus, in these animals a small portion of grafted cells followed the normal pathway for migration of neuroblasts generated in the SVZ to the MOB (Fig. 3a-b), but never reached beyond the rostral turn of the RMS. In contrast, in the group of rats subjected to stroke, the grafted cells showed a completely different migration pattern (Fig. 3c-d). In this case, the majority of transplanted cells left the RMS and migrated in the opposite direction toward the damaged area of the striatum. We also found that the mean distance which the grafted cells had migrated from the implantation site at 2 months after transplantation was longer in stroke-affected animals as compared to intact ones (795.4 ± 140.8 and 399.9 ± 44.96 μm, respectively). However, the maximum distance from the implantation site reached by the grafted cells was similar in both groups (Fig. 3e-f).

**Fig. 4:**
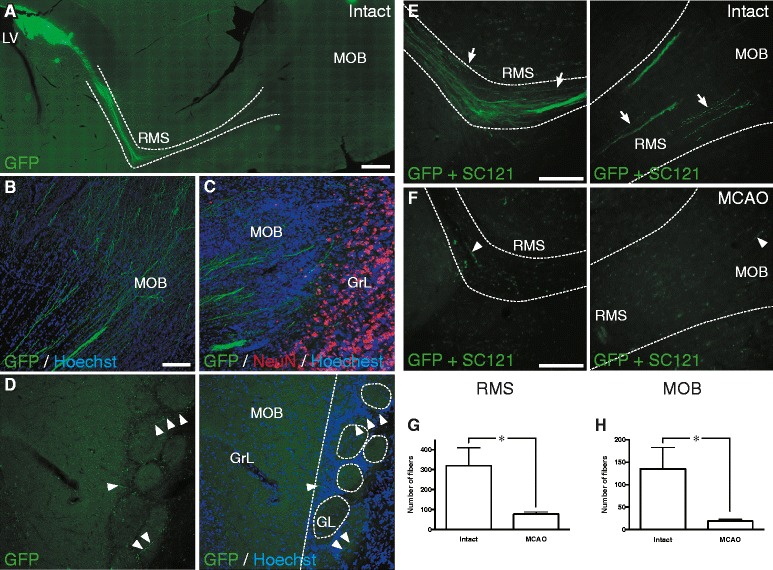
Stroke alters fiber outgrowth pattern of human skin-derived neural progenitors transplanted adjacent to SVZ. **a**-**c** Fluorescence photomicrographs showing fiber outgrowth from grafted cells (GFP+, *green*) 2 months after transplantation in intact and stroke-injured (MCAO) rats. In the intact brain, grafted cells send massive numbers of fibers through the RMS (**a**) reaching MOB (**b**) including granular layer (**c**) and glomerular layer (**d**; *arrowheads* depict GFP+ fibers). **e**-**h** Number of fibers crossing the rostral turn of the RMS (**e** and **f** *right*, and **g**) and reaching the MOB (**e** and **f** *left*, and **h**), from grafted cells 2 months after transplantation in intact (n = 6) and stroke-subjected (n = 7) rats. *Arrows* in (**e**) depict massive number of fibers in the intact brain and *arrowheads* in (**f**) depict rare fibers found in the stroke-subjected brains (MCAO). Data represent means ± SEM; *P < 0.05, two-tailed unpaired *t* test. *LV* lateral ventricle, *RMS* rostral migratory stream, *MOB* main olfactory bulb, *GrL* granular layer, *GL* glomerular layer. Scale bars represent 1 mm in (**a**), 50 μm in (**b**) also valid for (**c** and **d**) and 100 μm in (**e** and **f**)

Finally, we analyzed the fiber outgrowth from the grafted cells at 2 months after transplantation. In both groups of animals, the human iPSC-derived lt-NESCs transplanted into the RMS sent fibers to different areas of the brain, including some tangential projections to the cortex and the striatum, and others following white matter tracts like corpus callosum. When transplanted into the intact brain, the grafted cells also sent massive number of fibers through the RMS, reaching the granular and glomerular layers of the MOB (Fig. 4a-d). An average of more than 300 fibers per animal reached the rostral turn of the RMS, and more than 100 fibers arrived to the MOB (Fig. 4e and g-h). In contrast, in the stroke-injured animals, virtually no fibers reached the granular layer of the MOB and only very few the rostral turn of the RMS (Fig. 4f-g).

## Discussion

The results of the present study indicate that the presence of an ischemic lesion does not influence the survival, proliferation or differentiation of grafted cells after transplantation of human iPSC-derived lt-NESCs adjacent to a neurogenic area, i.e., the SVZ. In contrast, an ischemic injury causes marked changes in the migration and axonal projection patterns of the grafted cells. Thus, we found that in the intact brain, the grafted cells migrated a short distance from the implantation site along the RMS but sent massive number of fibers in the RMS reaching the MOB. In the stroke-damaged brain, on the other hand, the grafted cells migrated toward the ischemic lesion and the number of fibers sent through the RMS was dramatically reduced.

Transplantation of neural precursors in non-neurogenic areas of intact brains can lead to death of grafted cells within 1 week [8, 16]. Therefore, in order to allow graft survival also in the intact brain and assessment of the effect of an ischemic lesion, we implanted the human iPSC-derived lt-NESCs in the RMS, close to the SVZ. Neurogenic areas like the SVZ support survival and differentiation of grafted cells by molecular and cellular mechanisms, which are only partially understood [8]. We observed no significant difference in the survival, proliferation or differentiation of the grafted cells at 2 months after transplantation in stroke-damaged as compared to intact rat brain. In agreement, Jin and collaborators [16] showed that the presence of an ischemic damage after MCAO in rats did not affect the percentage of mouse embryonic neural precursors implanted in the striatum expressing phenotypic markers such as DCX, NeuN and GFAP.

The RMS displays the most substantial, long-distance neuronal migration in the mammalian postnatal brain. A wide range of extracellular factors guide neuroblasts formed in the SVZ along this migratory route, including cell adhesion/extracellular matrix (e.g., PSA-NCAM, integrins, and galectin-3) and axon guidance molecules (e.g., Slit/Robo, Netrin-1/Dcc, and Erb4/Neuregulin), growth factors (Sonic hedgehog, Nogo, and prokineticin-2) and neurotransmitters (GABA and glutamate) [17]. It has been reported that human fetal striatal neural stem cells and human iPSC-derived neural precursors grafted into the rat brain can migrate along this pathway, in some cases a few cells reaching the MOB [8, 18]. In contrast, we found that when the human iPSC-derived lt-NESCs were placed in the RMS in the intact rat brain, they only migrated a small distance from the injection site. Hypothetically, the timing of analysis after implantation could, at least partly, explain this difference in migration of the human iPSC-derived cells between our study (analysis at 2 months) and that of Major et al. ([8]; analysis at 3 months). Another possible explanation for the discrepancy in migratory behavior could be differences in the intrinsic chemotactic interaction between the grafted neural precursor cells and their neuronal progeny in the various transplants. Thus, limited neuronal migration in adult brain after transplantation was recently attributed to secretion of FGF2 and vascular endothelial growth factor (VEGF) from grafted neural precursors, which act as chemoattractants for neurons [19].

The presence of an ischemic lesion promoted the migration of the grafted cells toward the injured area, overcoming the regulatory signals in the intact brain. Similar targeted migration toward the ischemic cortical lesion has been found when human fetal neural stem cells were transplanted into the cerebral cortex of rats subjected to distal MCAO [20]. Also SVZ-derived neuroblasts deviate from their migratory path and are re-routed to sites affected by stroke-induced injury [21, 22], migrating closely associated with blood vessels [23]. The signals regulating the migration of the grafted human iPSC-derived lt-NESCs toward the ischemic lesion remain to be identified. Thus, it is unclear to what extent the same molecular and cellular mechanisms support the migration of neuroblasts in the intact and injured brain. Conceivably, the signals acting on the grafted human iPSC-derived lt-NESCs closely resemble those acting on the endogenous progenitors and neuroblasts after stroke. Molecular mechanisms directing these cells to the damaged area [24] include stromal cell-derived factor-1 (SDF-1) and monocyte chemoattractant protein-1 (MCP-1) in reactive astrocytes and activated microglia, acting on neural progenitors and new neuroblasts which express the respective receptors, CXCR4 and CCR2 [25–27]. Other molecules reported to be involved in this neuroblast migration are ostopontin acting on integrin ß1 receptor, matrix metalloproteinase 9 (MMP9), erythropoietin (EPO), and platelet-derived growth factor receptor beta (PDGFß) [28–31].

How the distance from the ischemic lesion influences the stroke-induced migratory behavior of the grafted cells is unclear. While some studies conclude that migration of transplanted cells toward the injury is not induced at distances longer than 1 mm [32], other studies describe long-distance (even inter-hemispheric) migration of murine stem cells implanted into the stroke-damaged rat brain [33, 34]. In our experiments, signals coming from the ischemic lesion were sufficient to induce the migration of the human iPSC-derived lt-NESCs from the RMS/SVZ in the direction of the injury, i.e., over a distance of 1 to 3 mm.

In the intact animals, we observed massive fiber outgrowth along the RMS from the grafted human iPSC-derived lt-NESCs. A similar outgrowth was previously shown from human tertocarcinoma cells transplanted into the striatum of adult rat brain [7], suggesting guidance cues in the adult brain that govern fiber outgrowth (e.g., ADAM21 metalloprotease; [35]). The same signals that regulate neuronal migration also mediate neurite outgrowth [36]. One example is netrin proteins, which are important regulators of axon guidance and cell migration, and whose receptor is present on neural precursors in the adult mouse and human SVZ and RMS [37]. Also Syndecan-3 has been reported to be crucial for radial migration and neurite outgrowth in the developing brain [38, 39]. This transmembrane proteoglycan is expressed in major axonal pathways and migratory routes of the brain, including the RMS. Interestingly, our results show that the presence of an ischemic damage in the striatum is able to abolish the fiber outgrowth from the grafted human iPSC-derived lt-NESCs through the RMS observed in the intact brain. This difference in the behavior of the grafted cells may be explained by their migration in the direction of the ischemic lesion. The typical unipolar morphology of the migrating neuroblasts is probably not compatible with sending long fibers to distant structures like MOB, located in the opposite direction.

## Conclusions

We show here that neurons generated from transplanted human skin-derived neural progenitors respond to signals regulating migration and fiber outgrowth in both the intact and stroke-injured brain. The signals from the ischemic lesion in the striatum seem to overcome those operating in the intact brain as evidenced by stroke causing a complete change in migratory and fiber outgrowth patterns of the graft-derived neurons. Taken together, our findings provide further evidence that transplantation of human skin-derived neural progenitors is a useful approach to supply new neurons to the adult brain. However, the occurrence of injured tissue strongly affects crucial parameters in the behavior of these cells, which will be important to consider in a potential, future clinical translation.

## Abbreviations

CCA: Common carotid artery
ECA: External carotid artery
EGF: Epidermal growth factor
FGF: Fibroblast growth factor
GFAP: Glial fibrillary acidic protein
GFP: Green fluorescent protein
ICA: Internal carotid artery
iPSCs: Induced pluripotent stem cells
KGA: Kidney-type glutaminase
lt-NESCs: Long-term neuroepithelial-like stem cells
MCAO: Middle cerebral artery occlusion
MOB: Main olfactory bulb
RMS: Rostral migratory stream
SD: Sprague-Dawley
SVZ: Subventricular zone
